# Exploring indicators of genetic selection using the sniffer method to reduce methane emissions from Holstein cows

**DOI:** 10.5713/ab.23.0120

**Published:** 2023-08-28

**Authors:** Yoshinobu Uemoto, Tomohisa Tomaru, Masahiro Masuda, Kota Uchisawa, Kenji Hashiba, Yuki Nishikawa, Kohei Suzuki, Takatoshi Kojima, Tomoyuki Suzuki, Fuminori Terada

**Affiliations:** 1 Graduate School of Agricultural Science, Tohoku University, Sendai 980-8572, Japan; 2 Gunma Prefectural Livestock Experiment Station, Maebashi 371-0103, Japan; 3 Niikappu Station, National Livestock Breeding Center (NLBC), Hidaka 056-0141, Japan; 4 Head office, National Livestock Breeding Center (NLBC), Nishigo 961-8061, Japan; 5 Institute of Livestock and Grassland Science, National Agriculture and Food Research Organization (NARO), Nasushiobara 329-2793, Japan; 6 Institute of Livestock and Grassland Science, NARO, Tsukuba 305-0901, Japan

**Keywords:** Commercial Dairy Farms, Enteric Methane Emission, Genetic Correlation, Heritability, Repeatability

## Abstract

**Objective:**

This study aimed to evaluate whether the methane (CH_4_) to carbon dioxide (CO_2_) ratio (CH_4_/CO_2_) and methane-related traits obtained by the sniffer method can be used as indicators for genetic selection of Holstein cows with lower CH_4_ emissions.

**Methods:**

The sniffer method was used to simultaneously measure the concentrations of CH_4_ and CO_2_ during milking in each milking box of the automatic milking system to obtain CH_4_/CO_2_. Methane-related traits, which included CH_4_ emissions, CH_4_ per energy-corrected milk, methane conversion factor (MCF), and residual CH_4_, were calculated. First, we investigated the impact of the model with and without body weight (BW) on the lactation stage and parity for predicting methane-related traits using a first on-farm dataset (Farm 1; 400 records for 74 Holstein cows). Second, we estimated the genetic parameters for CH_4_/CO_2_ and methane-related traits using a second on-farm dataset (Farm 2; 520 records for 182 Holstein cows). Third, we compared the repeatability and environmental effects on these traits in both farm datasets.

**Results:**

The data from Farm 1 revealed that MCF can be reliably evaluated during the lactation stage and parity, even when BW is excluded from the model. Farm 2 data revealed low heritability and moderate repeatability for CH_4_/CO_2_ (0.12 and 0.46, respectively) and MCF (0.13 and 0.38, respectively). In addition, the estimated genetic correlation of milk yield with CH_4_/CO_2_ was low (0.07) and that with MCF was moderate (−0.53). The on-farm data indicated that CH_4_/CO_2_ and MCF could be evaluated consistently during the lactation stage and parity with moderate repeatability on both farms.

**Conclusion:**

This study demonstrated the on-farm applicability of the sniffer method for selecting cows with low CH_4_ emissions.

## INTRODUCTION

Recently, there has been an increasing interest in reducing methane (CH_4_) concentrations in the air because of its strong impact on global warming, which is 28 times greater global warming potential than carbon dioxide (CO_2_) [1]. Enteric CH_4_ emissions from ruminants are a major contributor to greenhouse gas emissions in the agricultural sector [2,3], while dairy cows are major contributors to enteric CH_4_ emissions [4,5]. In addition, enteric CH_4_ emissions from cattle lead to lower animal productivity because they constitute approximately 2% to 12% of the gross energy intake (GEI) [6]. Therefore, reducing enteric CH_4_ emissions from cows benefits not only the environment but also farmers by reducing energy losses and improving dairy production system efficiency.

Several strategies to mitigate enteric CH_4_ emission from dairy cows include nutrition and feeding management, rumen modifiers, and genetic selection for increasing productivity and/or decreasing CH_4_ emission itself [7–10]. In particular, compared to alternative methods, such as non-genetic management approaches, genetic selection is a permanent and cumulative solution for improving the sustainability of the dairy industry while minimizing additional costs and labor for dairy farmers. Since enteric CH_4_ emission from dairy cows is a heritable and repeatable trait [7–9], the genetic selection of cows with low CH_4_ emissions has attracted attention for sustainable livestock production [10]. For the genetic selection of cows with low CH_4_ emissions, a practical and cost-effective measurement protocol is required to obtain phenotypic information on selection candidates or on animals of reference population for genomic evaluation.

The open-circuit respiration chamber method provides the most precise estimates of enteric CH_4_ emissions from cattle [6], but it incurs high costs and is impractical for measurements over a long period and of a large number of datasets. One of the strategies for measuring CH_4_ at low cost is the sniffer method, wherein the air near the animal’s nostrils is sampled through a fixed tube in a feed trough in an automatic milking system (AMS) [7,11–15]. In general, there are two types of sniffer methods: with or without CO_2_ measurement. The method without CO_2_ detection measures only the CH_4_ concentration in the sampled air and then applies its dilution factor to calculate the CH_4_ emissions [12]. The other method measures CH_4_ and CO_2_ concentrations simultaneously, calculates the CH_4_ to CO_2_ ratio (CH_4_/CO_2_), and CH_4_ emissions are obtained as the product of CH_4_/CO_2_ and the predicted CO_2_ values using body weight (BW), energy-corrected milk (ECM), and days of pregnancy, assuming a heat-producing unit (HPU) [11,13]. Studies have shown high correlation between CH_4_ emissions measured by the sniffer method and open-circuit respiration chambers [7]. The sniffer method with CO_2_ measurement has the advantage of being less influenced by the concentration of CH_4_ in the breath in the sampled air [13]. Furthermore, Suzuki et al [16] developed CH_4_ prediction equations, including CH_4_/CO_2_, BW, and ECM as independent variables, without considering the days of pregnancy and dry matter intake (DMI). However, when considering practical applications for measuring CH_4_ at the commercial farm level, BW, in particular, cannot be measured by most farmers, and thus, simple indicators are required for genetic selection to mitigate CH_4_ emissions.

Genetic improvements are important to reduce CH_4_ emissions in dairy cows without reducing productivity, such as milk yield. However, unfavorable genetic relationships between CH_4_ and milk yield have been reported in dairy cows [8,9]. Several phenotypes have been suggested for methane-related traits, including methane intensity (CH_4_ related to output) such as CH_4_ per ECM (CH_4_/ECM), methane yield (CH_4_ related to input) as methane conversion factor (MCF), and residual CH_4_ (RM) [17]. These methane-related traits obtained by the sniffer method may serve as indicators of CH_4_ mitigation breeding without reducing productivity in dairy cows. However, little is known about the impact of independent variables on predicting these methane-related traits in terms of environmental and genetic aspects. In addition, if CH_4_/CO_2_ can be utilized as a selection indicator, it will simplify evaluations without measuring other traits.

Therefore, this study aimed to evaluate whether CH_4_/CO_2_ and methane-related traits obtained by using the sniffer method can be used as indicators of genetic selection for reducing CH_4_ emissions in Holstein cows. In this study, a dataset was obtained from two farms. From the first farm dataset, we investigated the impact of the model with and without BW on the lactation stage and parity in predicting methane-related traits. The second farm dataset was used to estimate the genetic parameters for CH_4_/CO_2_ and methane-related traits. Third, the repeatability and environmental effects on these traits were compared in both farm datasets.

## MATERIALS AND METHODS

### Populations and data collection

Animal experiments were performed at the Gunma Prefectural Livestock Experiment Station, Gunma, Japan (Farm 1), and Niikappu station of the National Livestock Breeding Center (NLBC), Hokkaido, Japan (Farm 2), according to the animal care and use guidelines of the Gumma Prefectural Livestock Experiment Station and NLBC, respectively. The experiments at Farms 1 and 2 were evaluated and approved by the Gumma Prefectural Livestock Experiment Station (approval numbers: 2018.1(1), 2019.1(1), 2020.1(1), and 2021.1(1)) and NLBC (approval numbers: 30-17 and 31-04), respectively.

The daily milk yield of Holstein cows was recorded using Lely Astronaut A2 AMS (Lely, Maassluis, the Netherlands) on Farm 1 and DeLaval VMS Classic AMS (DeLaval International AB, Tumba, Sweden) on Farm 2. These records were obtained for seven consecutive days in 12 separate periods spanning October 2017 to March 2021 and seven separate periods from June 2018 to April 2020, on Farm 1 and Farm 2, respectively. During each sampling period, the daily milk yield was recorded for seven consecutive days, and the average daily milk yield (AMY, kg/d) was calculated. In each period, BW (kg) was measured only on Farm 1. The fat and protein percentages (Fat% and Pro%, respectively) were obtained during each measurement period from milk samples used in the most recent dairy herd improvement program of the Livestock Improvement Association of Japan (LIAJ) (Tokyo, Japan) or measured at the milk inspection station of milk marketing cooperatives in the Kinki region (Hyogo, Japan). The ECM (kg/d) was then calculated for each sampling period as follows [18];


$$\text{ECM}=\frac{\text{AMY}\times (376\times \text{Fat}\%+209\times \text{Pro}\%+948)}{3,138}$$

Cows were fed Partial Mixed Ration (PMR) nine and three times per day in Farm 1 and Farm 2, respectively, and the concentrate diet at every milking in the AMS in both farms. The percentage of the total digestible nutrient (TDN), crude protein (CP), and roughage on a dry matter basis in the PMR were 69% to 70%, 15% to 16%, and 40% to 60% in Farm 1, and 69% to 72%, 14% to 16%, and 41% to 44% in Farm 2, respectively. The percentage of the TDN and CP on a dry matter basis in the concentrate diet at AMS were 70% to 85% and 18% to 21% in Farm 1 and 86% and 24% in Farm 2, respectively.

### Gas measurement and methane-related traits

Gas measurement method previously reported by Oikawa et al [14] was used in this study. Briefly, the CH_4_ and CO_2_ emissions from the cows were measured during milking in each milking box of the AMS at the same time as the daily milk yield measurement period. The inlet for sampling was placed near the feed trough in the milking box. The air around the feed trough was sampled using a pump at a flow rate of 6.5 L/min. The concentrations of CH_4_ and CO_2_ in the sampled air were monitored and recorded using the Microportable Greenhouse Gas Analyzer (Model 909–0050; LGR Inc., CA, USA). The CH_4_ and CO_2_ concentrations of the sampled gas were logged at 1-s intervals. During each measurement period, these data were recorded daily for seven consecutive days. In this study, the corrected CO_2_ concentrations greater than 500 ppm were used after deducting background CO_2_ concentration from the sampled gas. Then, the average values of CH_4_ (ppm) and CO_2_ (ppm) per milking were estimated, and CH_4_/CO_2_ ratio was calculated.

The data for AMY, ECM, and CH_4_/CO_2_ were obtained from both farms, but BW was only recorded for cows in Farm 1 in this study. These traits were regarded as production traits in the present study. The four traits, CH_4_, CH_4_/ECM, MCF, and RM, were regarded as methane-related traits and were calculated using CH_4_/CO_2_ and ECM (CH4e, CH4e/ECM, MCFe, and RMe, respectively), and CH_4_/CO_2_, ECM, and BW (CH4eb, CH4eb/ECM, MCFeb, and RMeb, respectively) as independent variables using the prediction equations in Table 1, as shown by Suzuki et al [16] and Richardson et al [19]. The MCF, which is calculated by dividing CH_4_ by GEI, is the percentage of feed energy converted to CH_4_. The RM is estimated as the difference between the observed and predicted CH_4_ [17]. The equation for MCFeb was not presented by Suzuki et al [16] because they found that BW did not significantly affect MCF, but it was derived for our study using the dataset from Suzuki et al [16]. Richardson et al [19] reported that RM corrected for ECM was the most suitable indicator for dairy breeding programs; thus, RM corrected for ECM was used in our study. The Farm 1 dataset was used to evaluate the effect of the model with and without BW on the lactation stage and parity in predicting methane-related traits. For the Farm 2 dataset, only MCFe was calculated because MCF was not affected by BW (see Results section).

The CH_4_/CO_2_ of 556 records for 88 Holstein cows and 687 records for 277 Holstein cows were obtained from Farm 1 and Farm 2, respectively. The records and cows were then selected from both farms according to the following criteria: records collected between parity 1 and parity 3, records collected between 7 and 360 d in milk (DIM), and cows with phenotypic values for all traits and at least two records for each trait. In addition, cows with pedigree information were selected from Farm 2. In total, 400 records of 74 Holstein cows and 520 records of 182 Holstein cows were retained from Farm 1 and Farm 2, respectively. The number of Holstein cows per record per farm is shown in Table 2. These datasets were used for the statistical analyses.

### Statistical analyses

Statistical analyses for the Farm 1 dataset, which includes four production traits and eight methane-related traits, were performed using ASReml v4.1 software [20]. A linear mixed model was applied to estimate the fixed effects and variance components with standard errors (SEs) as follows:


(1) y=Xb+Za+e,

where **y** is a vector of the observations; **X** and **Z** are the known design matrices connecting observations to **b** and **a**, respectively; **b** is a vector of fixed effects related to the sampling periods (12 levels), lactation stage (12 levels of 30 days each from 7 to 30 days to 331 to 360 days), and parity (three levels); and **a** and **e** are vectors of the individual [ $a\sim N(0,\;\sigma_{\text{a}}^{2}I)$] and error effects [ $e\sim N(0,\;\sigma_{\text{e}}^{2}I)$], where $\sigma_{\text{e}}^{2}$ is the between-individual variance, $\sigma_{\text{a}}^{2}$ is the within-individual variance, and **I** is the identity matrix. The estimated repeatability (R) obtained by the model (1) is expressed as


$$\text{R}=\frac{{\hat{\sigma }}_{\text{a}}^{2}}{{\hat{\sigma }}_{\text{a}}^{2}+{\hat{\sigma }}_{\text{e}}^{2}},$$

where ${\hat{\sigma }}_{\text{a}}^{2}$ and ${\hat{\sigma }}_{\text{e}}^{2}$ are the estimated between- and within-individual variances, respectively.

For the Farm 2 dataset, including AMY, ECM, CH_4_/CO_2_, and MCFe, we used ASReml v4.1 software [20] to estimate fixed effects and (co)variance components (with SEs). Heritability and repeatability were estimated using the single-trait repeatability animal model as follows:


(2) y=Xb+Zu+Wpe+e,

where **y** is a vector of the observations; **X**, **Z**, and **W** are the known design matrices connecting observations to **b**, **u**, and **pe**, respectively; **b** is a vector of fixed effects related to the sampling periods (seven levels), lactation stage (12 levels of 30 days each from 7 to 30 days to 331 to 360 days), and parity (three levels); **u** is a vector of breeding values [ $u\sim N(0,\;\sigma_{\text{u}}^{2}A)$], where $\sigma_{\text{u}}^{2}$ is the additive genetic variance; **A** is the additive relationship matrix; **pe** is a vector of permanent environmental effects [ $\mathrm{pe}\sim N\left(0,\;\sigma_{\text{pe}}^{2}I\right)$], where $\sigma_{\text{pe}}^{2}$ is the permanent environmental variance; **I** is the identity matrix; and **e** is a vector of the error effects [ $e\sim N(0,\;\sigma_{\text{e}}^{2}I)$], where $\sigma_{\text{e}}^{2}$ is the error variance. The pedigrees were traced back to four generations, and 1,570 individuals were used in this study. The estimated heritability (h^2^) and R obtained by the model (2) are expressed as follows:


$$\begin{array}{l} {\text{h}}^{2}=\frac{{\hat{\sigma }}_{\text{u}}^{2}}{{\hat{\sigma }}_{\text{u}}^{2}+{\hat{\sigma }}_{\text{pe}}^{2}+{\hat{\sigma }}_{\text{e}}^{2}},\\ \text{R}=\frac{{\hat{\sigma }}_{\text{u}}^{2}+{\hat{\sigma }}_{\text{pe}}^{2}}{{\hat{\sigma }}_{\text{u}}^{2}+{\hat{\sigma }}_{\text{pe}}^{2}+{\hat{\sigma }}_{\text{e}}^{2}}, \end{array}$$

where ${\hat{\sigma }}_{\text{u}}^{2},{\hat{\sigma }}_{\text{pe}}^{2}$, and ${\hat{\sigma }}_{\text{e}}^{2}$ are the estimated additive genetic, permanent environmental, and error variance, respectively.

Genetic correlations were also estimated using a two-trait repeatability animal model based on the model (2); however, the random effects were different. Here, **u**, **pe**, and **e** are vectors of breeding values [**u**~***N***(**0**, **G_0_** ⊗ **A**)], permanent environmental effects [**pe**~***N***(**0**, **P_0_** ⊗ **I**)], and error effects [**e**~***N***(**0**, **R_0_** ⊗ **I**)] , respectively; **G_0_** is the additive genetic (co)variance matrix for an individual, **P_0_** is the permanent environmental (co)variance matrix for an individual, and **R_0_** is the error (co)variance matrix for an individual.

## RESULTS

### Impact of body weight on predicting methane-related traits

Table 3 shows the descriptive statistics of the phenotypes of Holstein cows from Farm 1 and Farm 2. The average values of methane-related traits using the model with BW were higher than those without BW in Farm 1. The average values of AMY and ECM in Farm 2 were higher than those in Farm 1, whereas the average values of CH_4_/CO_2_ and MCFe in Farm 2 were lower than those in Farm 1.

**Figure 1:**
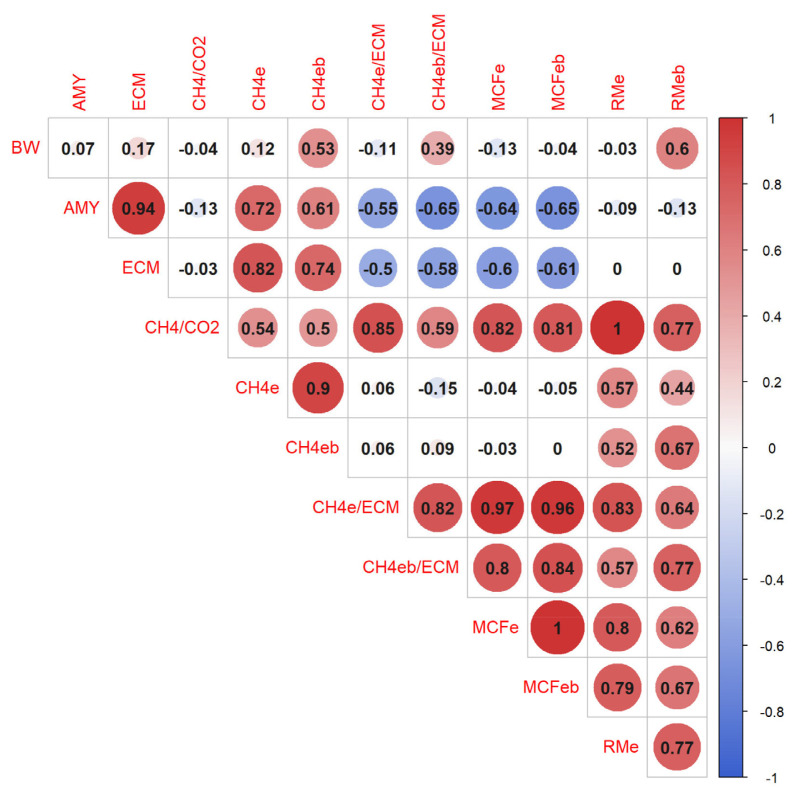
Correlation coefficients between production and methane-related traits. The red and blue colors of the circles indicate positive and negative correlations, respectively. Values marked with a background color are statistically significant (p<0.05). BW, body weight (kg); AMY, the average daily milk yield (kg/d); ECM, energy-corrected milk (kg/d); CH_4_/CO_2_, the ratio of methane to carbon dioxide (ppm/ppm). Abbreviations of methane-related traits are shown in Table 1.

Figure 1 shows the correlation coefficients between the production and methane-related traits in Farm 1. High correlation coefficients between methane-related traits were observed using the models with and without BW (ranging from 0.77 to 1.00). In particular, the correlation coefficients between MCFe and MCFeb were close to 1.00. The correlation coefficients of BW with CH_4_, CH_4_/ECM, and RM were moderately positive (ranging from 0.39 to 0.60) when BW was included in the model, while no correlation (correlation coefficients ranged from 0.11 to 0.12) was observed when BW was excluded from the model. The correlation coefficients of BW with CH_4_/CO_2_ and MCF were extremely low (ranging from −0.13 to −0.04). The correlation coefficient of AMY with CH_4_ was unfavorably moderate (0.72 for CH4e and 0.61 for CH4eb) and that with CH_4_/CO_2_ and RM was very low (ranging from −0.13 to −0.09). On the other hand, the correlation coefficients of AMY with MCF and CH_4_/ECM were moderately favorable (ranging from −0.65 to −0.55). The correlation coefficient of CH4eb with CH_4_/CO_2_ and MCFe was moderately favorable (0.50) and extremely low (0.00), respectively.

**Figure 2:**
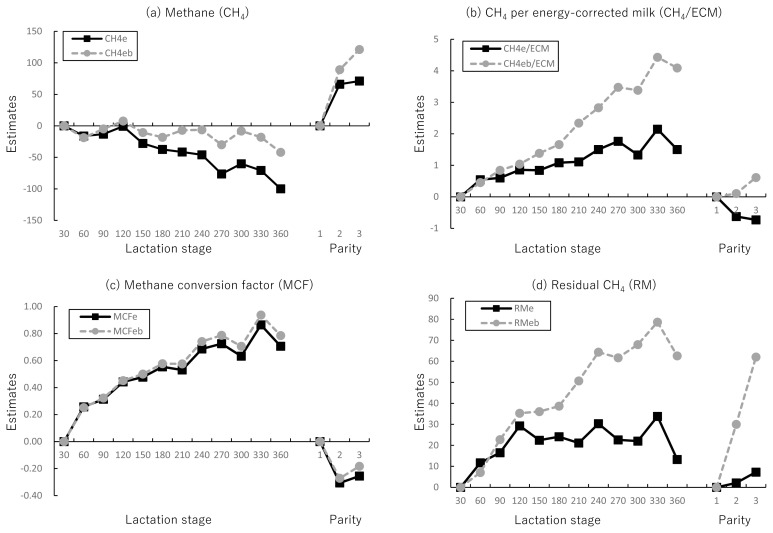
Estimates of methane-related traits during lactation stage and parity in Farm 1. The best linear unbiased estimate (BLUE) solutions for levels of fixed effect are shown in each trait. The estimates of the 7 to 30 days and first parity were fixed at zero. (a) Methane (CH_4_); (b) CH_4_ per energy-corrected milk (CH_4_/ECM); (c) methane conversion factor (MCF); (d) residual CH_4_ (RM). ECM, energy-corrected milk (kg/d). Abbreviations of methane-related traits are shown in Table 1.

For the environmental factors affecting methane-related traits, estimates from the lactation stage and parity are shown in Figure 2. During the lactation stage and parity, differences in estimates between the models with and without BW were observed for CH_4_, CH_4_/ECM, and RM. In particular, estimates of methane-related traits, excluding MCF, decreased in the latter period of the lactation stage and parity when BW was excluded from the model. In contrast, the estimates of MCF using the model with and without BW showed a similar trend during the lactation stage and parity.

The estimates of the variances and R for Farm 1 are shown in Table 4. R values were moderate to high for production traits (ranging from 0.42 to 0.73) and moderate for methane-related traits (ranging from 0.40 to 0.51). The R values for CH_4_, CH_4_/ECM, and RM were slightly increased when BW was included in the model, whereas no difference was observed in MCF.

### Genetic parameters and potential indicators

**Figure 3:**
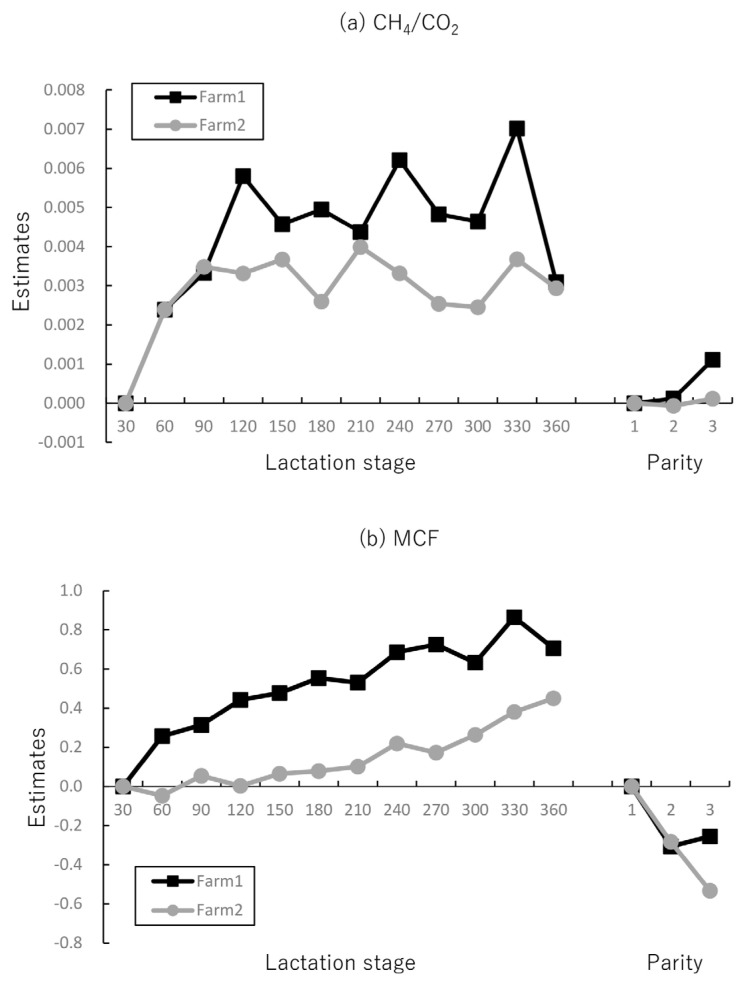
Estimates of (a) methane to carbon dioxide ratio (CH_4_/CO_2_) and (b) methane conversion factor using the model without body weight (MCFe) per farm during the lactation stage and parity. The best linear unbiased estimate (BLUE) solutions for levels of fixed effect are shown in each trait. The estimates of the 7 to 30 days and first parity were fixed at zero.

Since BW could not be measured on Farm 2, only MCFe from methane-related traits, which is independent of the presence or absence of BW in the model, was used in the analyses. Table 5 shows the h^2^ and R estimated for Farm 2. The h^2^ values were low and ranged from 0.10 to 0.15 for all traits, while the R values were moderate ranging from 0.38 to 0.47. The R values were similar to those of Farm 1. The estimated genetic correlation of AMY with CH_4_/CO_2_ was low (0.07), and that with MCF was moderate (−0.53). Regarding the environmental factors affecting CH_4_/CO_2_ and MCFe, the estimates for the lactation stage and parity in both farms are shown in Figure 3. Both these traits exhibited a similar trend in both the farms as the lactation stage and parity increased.

## DISCUSSION

### Indicators of genetic selection for low methane emissions from cows

Genetic selection for mitigating CH_4_ emissions from cows is an effective method for sustainable livestock production, and is also cost-effective because it produces permanent and cumulative changes in performance [8–10]. However, genetic selection requires CH_4_ data from a large number of samples measured at the commercial farm level. One way to overcome this problem of data measurement is to use predicted values for methane-related traits. Previous studies have estimated the genetic parameters from methane-related traits predicted using the model with DMI, milk yield, and BW [21–23], milk fat composition [24], and mid-infrared spectra of milk [25] in dairy cattle. However, predicted methane-related traits are expected values that are indirectly associated with feed efficiency and production traits. When considering genetic improvement for mitigating CH_4_ without depending on these traits, the actual values of CH_4_ are required. Therefore, it is necessary to develop a low-cost on-farm measurement method for CH_4_.

Recently, the sniffer method has been reported for measuring CH_4_, and the possibility of genetic improvement using this method has been reported [8,9]. This method can record a large number of CH_4_ readings at the commercial farm level [12–14]. The sniffer method can measure CH_4_/CO_2_, but phenotypic values, such as BW and ECM, are needed to evaluate methane-related traits [16]. To apply methane-related traits for genetic selection to reduce CH_4_ emissions from dairy cows, it is necessary to identify indicators for the prediction that are unaffected by independent variables and have a favorable relationship with productivity. Here, we evaluated whether CH_4_/CO_2_ and methane-related traits obtained using the sniffer method can be used as indicators of genetic selection from an environmental and genetic perspective.

### Impact of body weihgt on predicting methane-related traits

The impact of the model with and without BW on the lactation stage and parity in predicting methane-related traits was investigated. The findings of this study indicate that the inclusion of BW in the prediction models for CH_4_, CH_4_/ECM, and RM resulted in differences in estimates, especially in the latter stages of lactation and parity. In contrast, no difference was found in MCF, regardless of the presence or absence of BW in the model. Next, we investigated the differences between Farm 1 and Farm 2 for CH_4_/CO_2_ and MCFe and found that these two traits exhibited comparable trends as the lactation stage and parity increased. These results suggest that CH_4_/CO_2_ and MCFe can be consistently evaluated during the lactation stage and parity, even if BW cannot be estimated.

There are few reports on the CH_4_ trend during parity, but many studies have indicated that during the lactation stage, CH_4_ tends to increase in the first few weeks, followed by a constant or gradual decrease in the latter period [26–29]. These results are similar to the trend of CH4eb, which was consistent in the latter period. On the other hand, CH4eb did not increase in the early stage of lactation. There is a possibility of overestimation of CO_2_ emissions in the early period, in which body-fat mobilization occurs frequently, resulting in negative energy balance and increased apparent feed efficiency [16,30]. Actually, the CH_4_ emission measured by the sniffer method [11,13] does not consider the variation in energy-utilization efficiency and body-fat mobilization caused by the individual feed efficiency and lactation stage [31]. Thus, caution should be exercised in determining CH_4_ emissions using CH_4_/CO_2_ by the sniffer methods.

There is limited information on the trend of CH_4_/CO_2_, CH_4_/ECM, MCF, and RM during the lactation stage and parity; however, CH_4_/ECM and RM exhibited similar trends to CH_4_ in our study, that is, the values decreased in the latter stage of lactation and parity when BW was excluded from the model. The results indicated that it is important to include BW in the estimation model when predicting CH_4_, CH_4_/ECM, and RM.

### Genetic parameters and potential indicators

Genetic analyses showed that the heritability estimates for CH_4_/CO_2_ and MCF were found to be low on Farm 2. However, their repeatability was estimated to be moderate on both farms. Previous studies have shown that estimated heritability and repeatability are low in CH_4_/CO_2_ [29,32] and moderate in methane yield, such as the ratio of CH_4_ to DMI (CH_4_/DMI) [19,33], but little is known about MCF, which is one of the methane yields. While the heritability estimates for CH_4_/CO_2_ were low, high SEs of estimated heritability for all traits were observed because of the limited sample size. It should be noted that the small sample size may bias the heritability estimates for these traits in this study, and further studies are needed to estimate heritability accurately by increasing the number of cows with records. In contrast, the repeatability estimates for CH_4_/CO_2_ and MCF were similar and moderate in both the farms and their SEs were low, suggesting the applicability of the sniffer method for the selection of cows with low CH_4_ emissions at the farm level.

In this study, the genetic correlation of AMY with CH_4_/CO_2_ and MCF was low and favorably moderate, respectively. The genetic correlation between CH_4_/DMI and milk yield has been reported to be low [33], but little is known about the genetic relationship of milk yield with CH_4_/CO_2_ and MCF. In this study, the genetic correlations of CH_4_ with CH_4_/CO_2_ and MCF could not be estimated because of the lack of BW data on Farm 2. However, the results from Farm 1 showed that the phenotypic correlation coefficient of CH4eb with CH_4_/CO_2_ and MCFe was moderate and extremely low, respectively. Therefore, further study is needed to evaluate the genetic correlations of CH_4_ with CH_4_/CO_2_ and MCF for the applicability of these traits as selection indicators. Caution should be exercised in the genetic selection of cows with low CH_4_ emissions using CH_4_/CO_2_. Improving feed efficiency reduces CO_2_ emissions, and thus increases CH_4_/CO_2_. In contrast, improved feed efficiency increases milk yield and thus increases CO_2_ production. Consequently, efficient cows (i.e., high milk yield and high feed efficiency) produce less heat (i.e., CO_2_) per unit of metabolic BW and ECM, overestimating CH_4_ production [15,31]. Therefore, genetic improvement in both CH_4_ and milk yield should be considered simultaneously when using CH_4_/CO_2_.

### Measurement of methane traits using the sniffer method

In this study, moderate repeatability for CH_4_/CO_2_ was estimated using the sniffer method in the two farm populations. In addition, a similar trend of CH_4_/CO_2_ was observed as the lactation stage and parity increased in both farms. Thus, the sniffer method was found to be a reliable method for measuring CH_4_/CO_2_ in dairy cows at the commercial farm level. The sniffer method is often criticized for its high experimental variation because the dilution of the exhaled air differs in animals due to the repeatability of the head position relative to the sampling tube during milking [8,14,31]. However, the sniffer method, which measures CH_4_ and CO_2_ concentrations simultaneously, provides accurate CH_4_/CO_2_ under certain CO_2_ concentrations by eliminating the effect of low breath concentrations [13,14]. A sampled gas containing a certain concentration of CO_2_ (greater than 500 ppm) was adopted in this study. The results revealed that CH_4_/CO_2_ is a repeatable trait with no adverse effects on milk yield, suggesting that the seletion of cows with low CH_4_ emissions using the sniffer method could be effective at the farm level.

### Utilization of the sniffer method in breeding programs

In this study, the heritability estimates for CH_4_/CO_2_ and MCF were low, and these results were comparable to those from previous studies [29,32]. Heritability estimates suggest that genetic selection based on CH_4_/CO_2_ and MCF alone may not effectively reduce enteric CH_4_ emissions in dairy cows. However, the genetic approach that combines increased productivity with reduced CH_4_ emissions may effectively reduce enteric CH_4_ emissions from dairy cows. Knapp et al [10] suggested that genetic improvement for overall productivity has the potential to reduce CH_4_/ECM by 9% to 19% in dairy cows. In addition, an integrated approach that combines genetic improvement with environmental management strategies will also be necessary to achieve a significant reduction. Knapp et al [10] also suggested that the reduction of 15% to 30% in CH_4_/ECM can be achieved in dairy production systems through the combination of genetic selection, feed efficiency and nutrition, rumen modifiers, and other management approaches. Therefore, the genetic selection for CH_4_/CO_2_ and MCF alone has a small impact on the reduction of enteric CH_4_ emissions from dairy cows, and integrated approaches should be considered in breeding programs for a significant reduction of enteric CH_4_ emissions from dairy cows.

Since the repeatability estimates for CH_4_/CO_2_ and MCF were moderate in this study, it was possible to identify individuals with consistently low CH_4_ emissions over time by utilizing repeatability information. Repeatability is the phenotypic correlation of repeated measures [34] and provides information about the consistency of traits across multiple measurements within an individual over time. The difference between repeatability and heritability is caused by a permanent environmental effect that consists of non-additive genetic and other environmental factors [34]. The permanent environmental effect is not an additive genetic effect, but farmer and breeding programs can leverage the effect to identify individuals that consistently exhibit low CH_4_ emissions over time. In this case, a hypothetical scenario in which dairy farmers aim to reduce CH_4_ emissions in their herd through genetic selection can be assumed as a strategy to apply repeatability information. The sniffer method enables the farmer to measure CH_4_/CO_2_ in a large number of cows and identify cows that consistently exhibit favorable methane-related traits. Through selective breeding, the farmer can gradually develop a herd with a lower propensity for CH_4_ emissions without compromising productivity in the long run. Thus, the sniffer method can be practically applied and repeatability estimates can be utilized in the selective breeding of cows with low CH_4_ emissions.

## CONCLUSION

For genetic selection aimed at reducing CH_4_ emissions from dairy cows, it is necessary to identify selection parameters that can be measured cost-effectively in a large number of samples on commercial farms. This study investigated whether CH_4_/CO_2_ and methane-related traits obtained from the sniffer method can be used as indicators of genetic selection to reduce CH_4_ emissions in Holstein cows. Our results indicated that CH_4_/CO_2_ and MCFe could be evaluated reliably during the lactation stage and parity and served as indicators of dairy cows with low CH_4_ emissions without reducing their productivity. For genetic selection of cows with low CH_4_ emission, the low heritability and moderate repeatability estimates of CH_4_/CO_2_ and MCFe indicated that genetic selection based on these traits alone may not effectively reduce enteric CH_4_ emissions in dairy cows, however, by utilizing repeatability information, it may be possible to identify the cows with consistently low CH_4_ emissions over time. Our results are based on a limited sample size, but the findings from this study suggest that the sniffer method can be effectively employed at the farm level for the selection of cows with low CH_4_ emissions. Further study is needed to estimate the heritability of CH_4_/CO_2_ and MCF accurately and the genetic relationships of CH_4_ with CH_4_/CO_2_ and MCF by considering larger sample sizes.

## Figures and Tables

**Table 1:** Definitions of eight methane-related traits in Holstein cows

Traits	Abbreviations	Equations^1)^
Methane (CH_4_, L/d)	CH4e	−248+10.5×ECM+5,169×CH_4_/CO_2_
	CH4eb	−507+0.536×BW+8.76×ECM+5,029×CH_4_/CO_2_
CH_4_ per ECM (CH_4_/ECM, L/kg)	CH4e/ECM	CH4e/ECM
	CH4eb/ECM	CH4eb/ECM
Methane conversion factor (MCF, J/100J)^2)^	MCFe	2.91–0.0498×ECM+51.0×CH_4_/CO_2_
	MCFeb	2.546+0.000742×BW−0.0521×ECM+50.83×CH_4_/CO_2_
Residual CH_4_ (RM, L/d)	RMe	CH4e−(10.26×ECM+153.46)
	RMeb	CH4eb−(9.48×ECM+224.32)

BW, body weight (kg); ECM, energy-corrected milk (kg/d); CH_4_/CO_2_, ratio of CH_4_ to carbon dioxide (ppm/ppm); b, the linear regression coefficient of CH_4_ on ECM.

1) Equations of CH_4_ and MCF were previously developed and reported by Suzuki et al [16] and those of RM were developed using the records on Farm 1 based on Richardson et al [19].

2) J/100J, Joules (J) per 100 J of gross energy intake.

**Table 2:** The number of Holstein cows per record per farm

N of records	Farm 1	Farm 2
2	15	81
3	7	68
4	8	19
5	4	6
6	15	8
7	8	0
8	10	0
9	4	0
10	2	0
15	1	0
Total	74	182

**Table 3:** Descriptive statistics of production and methane-related traits in Holstein cows from the two farms

Traits	Mean	SD	Min	Max
Farm 1 (74 cows with 400 records)				
BW (kg)	697	78	475	880
AMY (kg/d)	33.9	8.6	10.9	61.5
ECM (kg/d)	34.2	7.5	14.6	54.8
CH_4_/CO_2_ (ppm/ppm)	0.076	0.010	0.050	0.110
CH4e (L/d)	504.15	93.73	251.62	766.47
CH4eb (L/d)	548.44	96.69	314.49	780.73
CH4e/ECM (L/kg)	14.96	2.00	10.81	22.49
CH4eb/ECM (L/kg)	16.38	2.68	10.98	25.92
MCFe (J/100J)^1)^	5.09	0.66	3.08	7.13
MCFeb (J/100J)^1)^	5.15	0.66	3.06	7.13
RMe (L/d)	0.00	53.10	−135.79	171.24
RMeb (L/d)	0.00	65.21	−159.13	162.41
Farm 2 (182 cows with 520 records)				
AMY (kg/d)	40.6	8.0	22.2	66.5
ECM (kg/d)	40.5	6.9	23.9	61.5
CH_4_/CO_2_ (ppm/ppm)	0.062	0.007	0.048	0.079
MCFe (J/100J)^1)^	4.07	0.47	2.80	5.22

SD, standard deviation; BW, body weight; AMY, average daily milk yield; ECM, energy-corrected milk; CH_4_/CO_2_, ratio of methane to carbon dioxide; RM, residual CH_4_; MCF, methane conversion factor. Abbreviations of methane-related traits are shown in Table 1.

1) J/100J, Joules (J) per 100 J of gross energy intake.

**Table 4:** Estimates of variance and repeatability for production and methane-related traits in Holstein cows from Farm 1

Traits	V_b_^1)^		V_w_^1)^		R^1)^	
BW	2,343.08	(444.61)	864.29	(70.90)	0.73	(0.04)
AMY	23.89	(5.02)	26.81	(2.17)	0.47	(0.06)
ECM	16.65	(3.64)	22.60	(1.83)	0.42	(0.06)
CH_4_/CO_2_	0.000027	(0.000006)	0.000037	(0.000003)	0.43	(0.06)
CH4e	2,657.02	(578.87)	3,257.45	(264.83)	0.45	(0.06)
CH4eb	2,773.91	(593.99)	2,879.56	(234.87)	0.49	(0.06)
CH4e/ECM	1.10	(0.24)	1.53	(0.12)	0.42	(0.06)
CH4eb/ECM	2.06	(0.44)	2.65	(0.21)	0.44	(0.06)
MCFe	0.11	(0.02)	0.16	(0.01)	0.40	(0.06)
MCFeb	0.11	(0.02)	0.17	(0.01)	0.40	(0.06)
RMe	733.87	(164.54)	984.29	(80.09)	0.43	(0.06)
RMeb	1,188.39	(249.66)	1,133.86	(92.48)	0.51	(0.06)

V_b_, between-individual variance; V_w_, within-individual variance; R, repeatability; BW, body weight; AMY, average daily milk yield; ECM, energy-corrected milk; CH_4_/CO_2_, ratio of methane to carbon dioxide. Abbreviations of methane-related traits are shown in Table 1.

1) Standard errors are shown in parentheses.

**Table 5:** Estimates of genetic parameters for production and methane-related traits in Holstein cows from Farm 2^1)^

Traits	Genetic variance		Heritability		Repeatability		Genetic/phenotypic correlations^2)^							

							AMY		ECM		CH_4_/CO_2_		MCFe	
AMY	6.2	(6.83)	0.15	(0.17)	0.47	(0.05)			0.76	(0.24)	0.07	(0.80)	−0.53	(0.46)
ECM	3.1	(4.08)	0.10	(0.13)	0.39	(0.05)	0.91	(0.01)			−0.01	(0.93)	−0.78	(0.35)
CH_4_/CO_2_	0.0000019	(0.000002)	0.12	(0.14)	0.46	(0.05)	0.12	(0.05)	0.19	(0.05)			0.63	(0.55)
MCFe	0.013	(0.01)	0.13	(0.13)	0.38	(0.05)	−0.73	(0.03)	−0.77	(0.02)	0.49	(0.04)		

AMY, average daily milk yield; ECM, energy-corrected milk; CH_4_/CO_2_, ratio of methane to carbon dioxide; MCFe, methane conversion factor using the model without body weight.

1) Standard errors are shown in parentheses.

2) Upper diagonal is genetic correlation, lower diagonal is phenotypic correlation.
